# Supplementary figures and images for: Nucleoplasmic lamin C rapidly accumulates at sites of nuclear envelope rupture with BAF and cGAS
Source: J Cell Biol. 2022 Oct 27;221(12):e202201024. doi: 10.1083/jcb.202201024 (PMC9617480; doi:10.1083/jcb.202201024)

Source data for Fig. 6

**B**

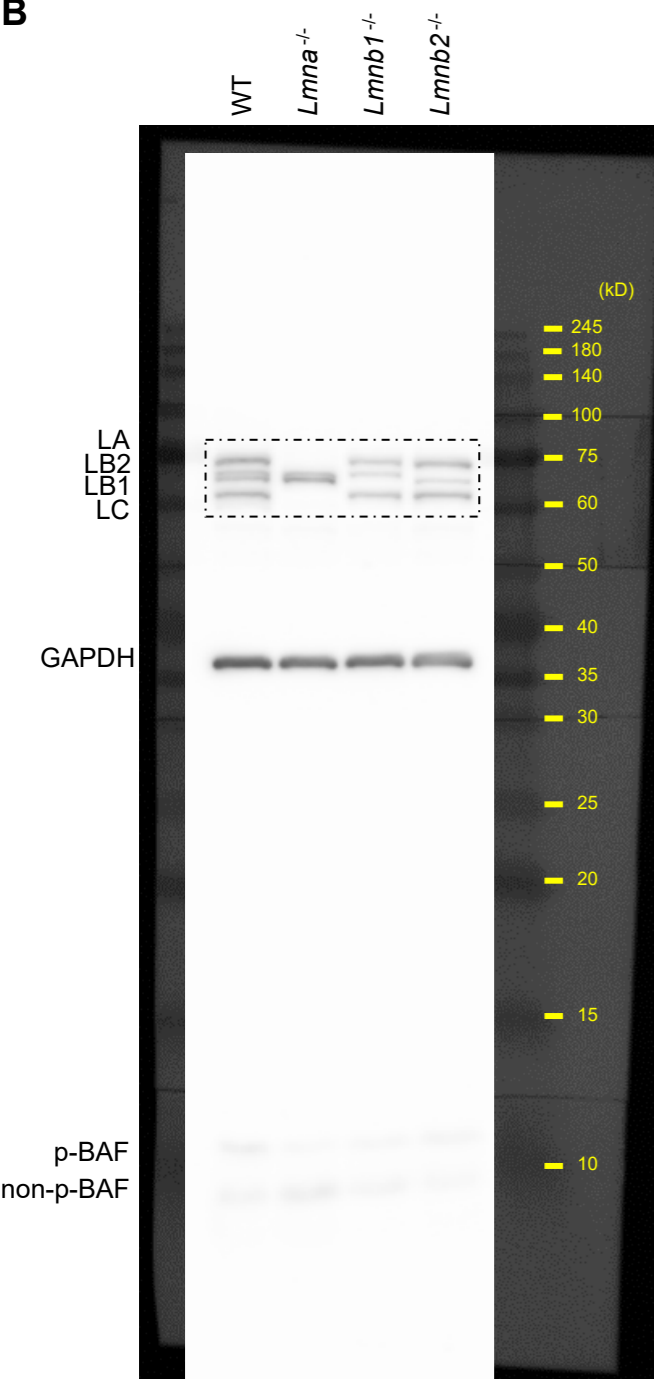

**D**

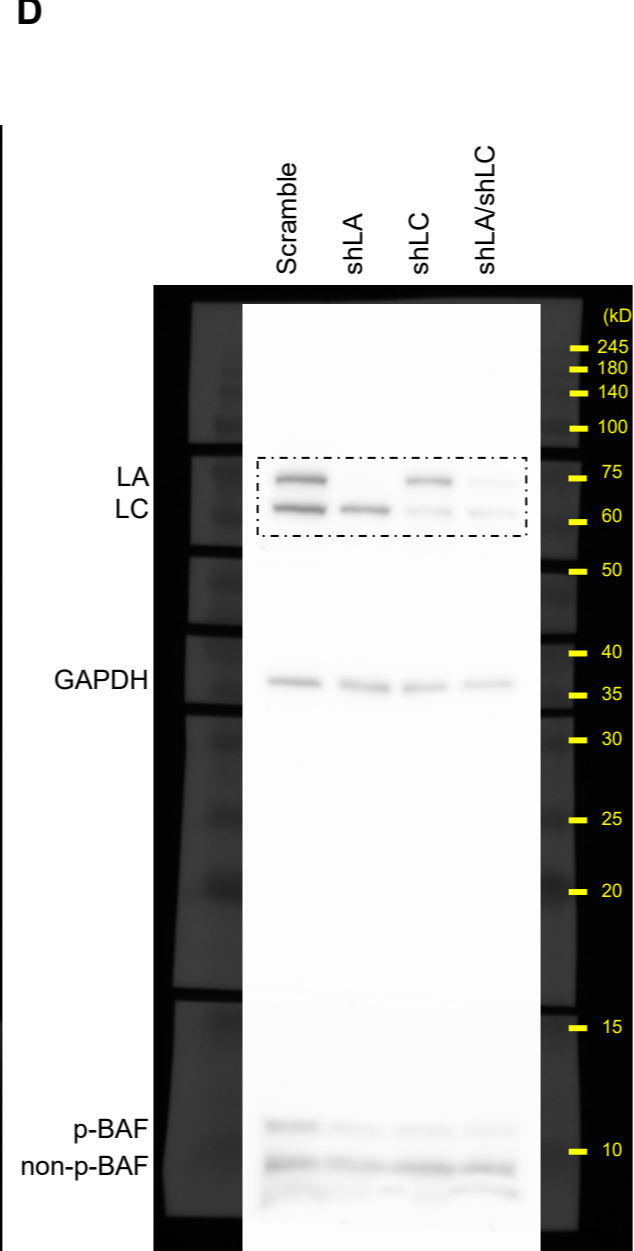

**F**

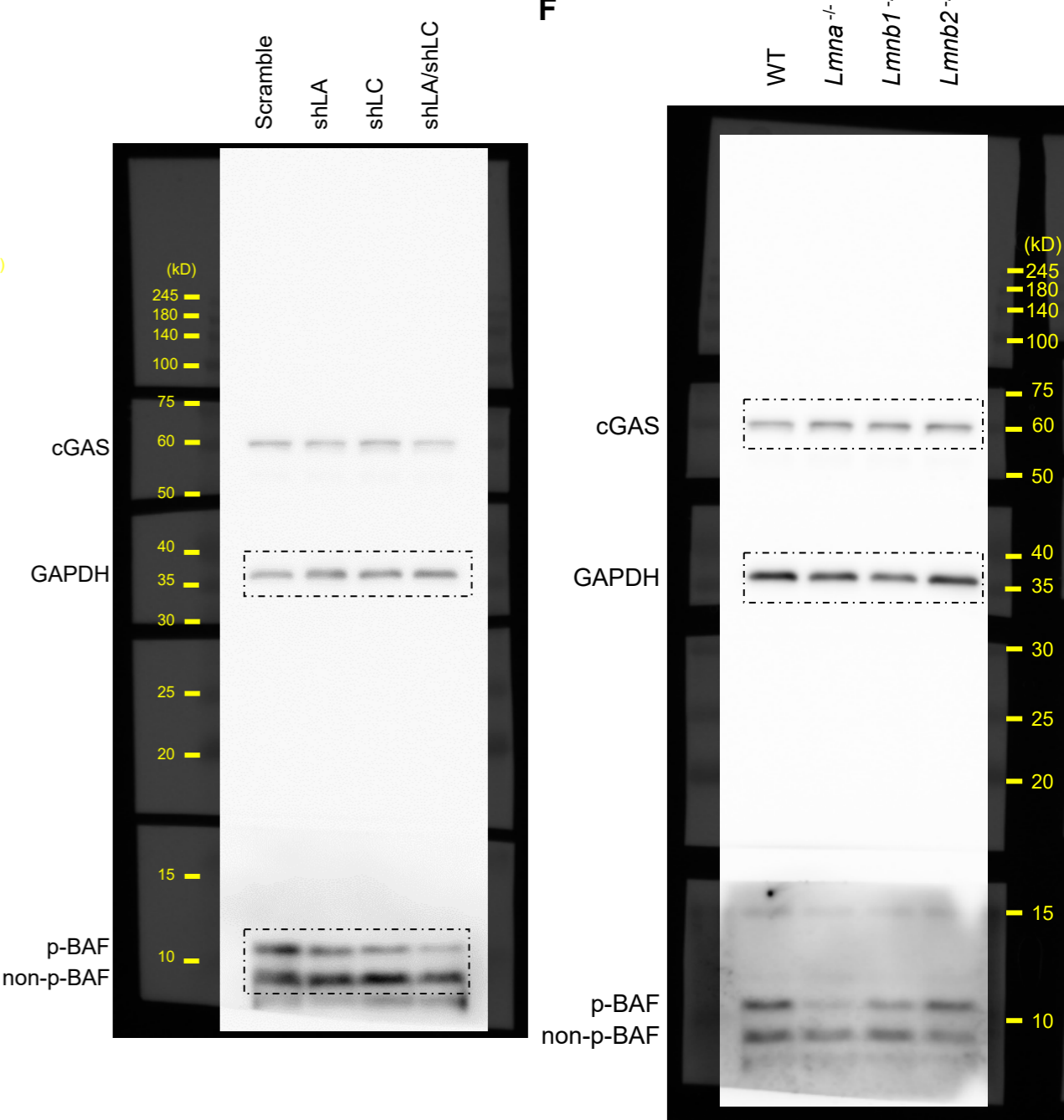

Supplement: SourceData F6 — is the source file for Fig. 6. [file JCB_202201024_SourceDataF6.pdf]

Source data for Fig. 9

A

*Lmna*<sup>-/-</sup> MEFs

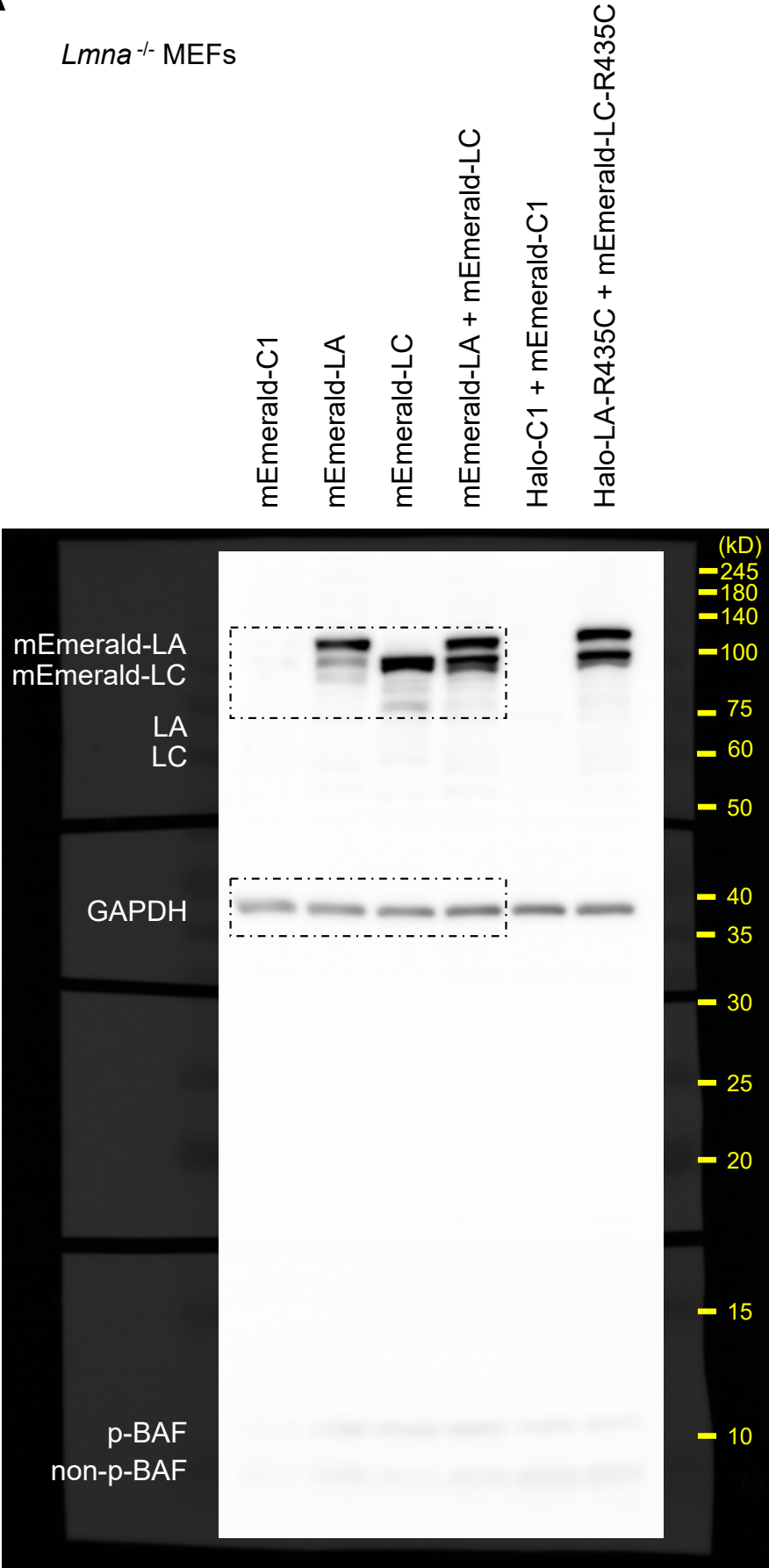

Supplement: SourceData F9 — is the source file for Fig. 9. [file JCB_202201024_SourceDataF9.pdf]

Source data for Fig. S2

B

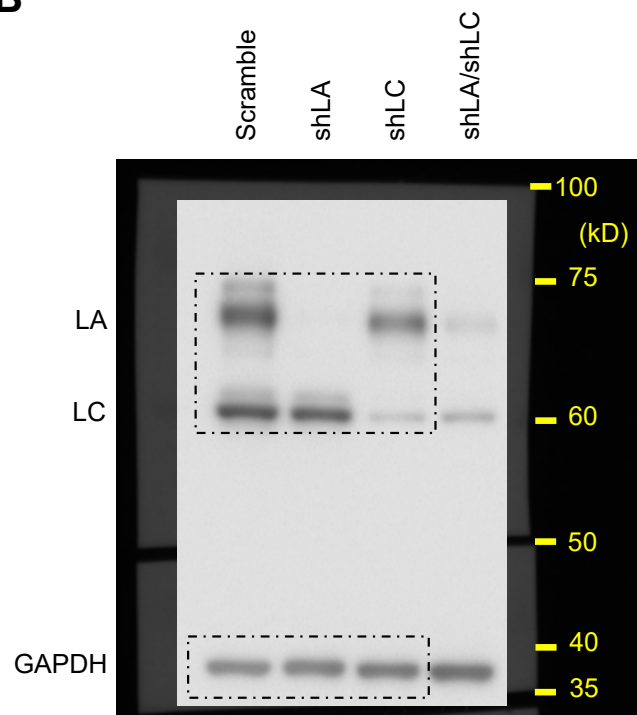

Supplement: SourceData FS2 — is the source file for Fig. S2. [file JCB_202201024_SourceDataFS2.pdf]

Source data for Fig. S4

C Short exposure

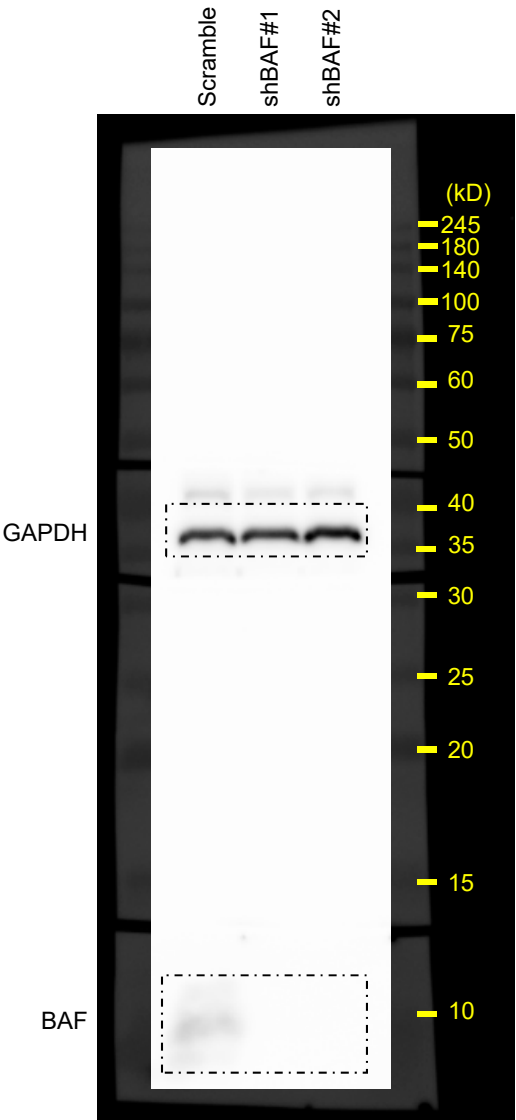

Long exposure

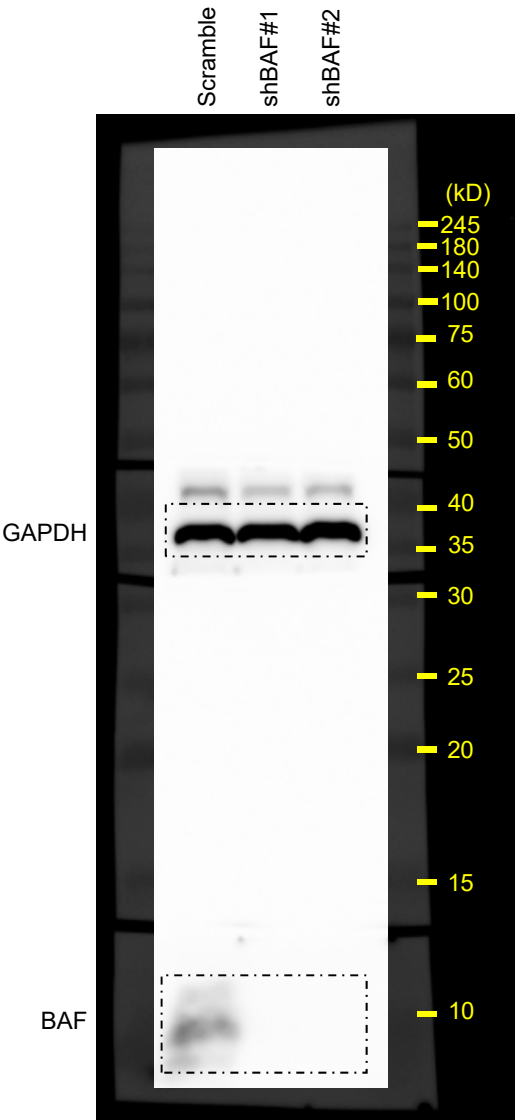

Supplement: SourceData FS4 — is the source file for Fig. S4. [file JCB_202201024_SourceDataFS4.pdf]
